# A phenomenological study on recurrent teenage pregnancies in effutu municipality- Ghana.the experiences of teenage mothers

**DOI:** 10.1186/s12889-023-15074-3

**Published:** 2023-02-01

**Authors:** Agartha Afful Boateng, Charles Owusu-Aduomi Botchwey, Bruce Afeti Adatorvor, Michael Afari Baidoo, Dorothy Serwaa Boakye, Richard Boateng

**Affiliations:** 1 Department of Health Administration and Education, Faculty of Science Education, University of Education, Post Office Box 25, Central Region Winneba, Ghana; 2 Volta Regional Hospital, Post Office Box 27, Hohoe, Volta Region Ghana

**Keywords:** Recurrent teenage pregnancy, Causes, Teenage mothers, Challenges

## Abstract

**Background:**

Generally, recurrent teenage pregnancies are public health menaces that impede the quality of life of teenage mothers, their offspring, and society as a whole. However, there is paucity of information regarding factors influencing this social issue especially, in developing countries where Ghana is no exception. Moreover, this menace has been least investigated from the perspective of the teenager with multiple pregnancies. Hence, this study aimed at identifying the factors influencing recurrent teenage pregnancies and the challenges confronted by these teenage mothers.

**Method:**

This study is a phenomenological qualitative study that was conducted in the Effutu Municipality in the Central Region of Ghana. Employing convenience and snowball sampling, 40 participants who were residents of the study area, had a child each, and were pregnant at the time of the study were included. Other participants included teenage mothers who had at least two (2) children. A face-to-face in-depth interview with the help of an interview guide was conducted. Proceedings were recorded, transcribed, and analysed using thematic analysis. Quotations were used in the result presentation.

**Results:**

The results of the study revealed that factors influencing recurrent teenage pregnancies are multifactorial. It includes peer pressure, parental neglect, poverty, living with a partner, and inadequate knowledge of family planning. The teenager with recurrent pregnancy is confronted with financial difficulties and is faced with stigmatisation in the society where she finds herself.

**Conclusion:**

To this effect, it is important to intensify education on family planning and good parental practices among parents with teenage mothers while providing a similar form of sensitization for members of the society about the harmful effects of stigmatisation on the teenage mother and her children. Again, a social support network for teenagers with recurrent pregnancies could be formed to help curb this public health menace.

## Introduction

In Ghana, 76 per 1000 births are born by teenage mothers between the age ranges of 15 to 19 years old [1]. Globally, 49 per 1000 births are born by mothers aged 15 to 19 years old [2]. Thus, in Ghana, the prevalence of teenage pregnancy within the aforementioned age range is almost one-fifth of the global prevalence. The central region has been of major concern to both governmental and non-governmental organizations due to the upsurge in teenage pregnancies [3]. The central region where Effutu municipality is located has about 21.3% of females between 15 to 19 years old bearing children [1, 4]. In Effutu municipality- a suburb of the central region, adolescent pregnancy has become a great deal of public concern [5]. A media report on teenage pregnancy in the Effutu municipality stated that within the 2013/2014 academic year, 36 teenage pregnancies were recorded among adolescents of school-going age. This number increased to 43 in the 2014/2015 academic year. Victims were forced to drop out of school, subsequently affecting their education [6]. Teenage pregnancy impedes a country’s ability to achieve the Sustainable Development Goals 3.7 and 5.2, thus, ensuring universal access to sexual and reproductive healthcare services, and achieving gender equality and empowering all women and girls by 2030 respectively [7–9].

Recurrent teenage pregnancy occurs when there is more than one pregnancy before the age of 20 amongst females [10]. Teenage mothers have about 30%, and 50% chances of becoming pregnant again within a year, and two years after the first pregnancy respectively [11, 12]. The care of an additional family member demands extra resources, further deteriorating the quality of life of the mother and her offspring [13]. Also, recurrent pregnancy within short intervals predisposes the girl to poor pregnancy outcomes and reduces her ability to access quality education or vocational training [14]. Her chances of acquiring gainful employment become a difficult task, resulting in a possible vicious cycle of poverty in their respective families. Factors influencing recurrent teenage pregnancies are multifactorial. They include poverty, young age of first pregnancy, adolescence marriage, being a child of a teenager, and poor attitude towards family planning among teenagers. [10, 11, 14, 15].

There is an extensive pile of literature on factors influencing teenage pregnancy and challenges confronted by teenage mothers in Ghana [16–20], however, most of these studies focused on single births [21] and used quantitative methods. A quantitative study does not provide an in-depth understanding of this social issue. Moreover, there is a paucity of information on the causes of recurrent teenage pregnancy in developing countries and the challenges such mothers are usually confronted with [14]. A study conducted in Accra, a city in Ghana among 33 teenagers with repeated pregnancies to ascertain the challenges confronting them revealed that educational, financial, health, and stigmatization were the challenges they faced [21]. The authors did not investigate the causes of recurrent teenage pregnancy from the perspective of teenage mothers. The causes and challenges faced by teenage mothers with recurrent pregnancies may have some cultural, socio-demographic, and ethnic variations [22]. Thus, there is the need to conduct studies in various geographical areas to identify the specific causes and challenges faced by teenagers with recurrent pregnancies to assist develop and implement community-centered interventions to tackle this public health menace. Furthermore, adolescent pregnancy tends to be higher among those with low economic status [9], the central region has been identified as among the poorest regions in Ghana, and this predisposes teenagers in the region to teenage pregnancy. Effutu municipality is inhabited by people from diverse socio-economic backgrounds coupled with the ascendency of teenage pregnancy in the municipality [6], these make the municipality appropriate to be selected to represent the Central region as a whole to investigate the menace.

The specific objectives of this study was to explore the factors influencing recurrent teenage pregnancies and challenges confronted by these teenage mothers in the Effutu Municipality of the Central Region of Ghana. Hence, the study sought to explore two primary questions: 1) What are the factors influencing the occurrence of recurrent teenage pregnancy? 2) What are the challenges faced by teenagers with recurrent pregnancies?

## Theoretical framework

The socio-ecological model was adapted to explain the multiple factors influencing recurrent teenage pregnancy [23] and the challenges comfronted by teenagers with recurrent pregnancy. The model indicates that people interact with different social and ecological factors at different levels, which influence their experiences and behaviors [24]. These levels consist of individual, interpersonal, and environmental factors [23]. At the individual level, the teenagers’ characteristics such as knowledge of contraceptives, use of contraceptives, early marriage, poverty, and low level of education [14, 25] may influence the occurrence of recurrent pregnancy and would be confronted by challenges such as dropping-out of school, financial hardship, and depression [21, 26]. At the interpersonal level, teenager’s closest social circle such as peer pressure, living with a partner, parental support, and higher perceived parental monitoring [14, 25] may influence the occurrence of recurrent teenage pregnancy and the teenager may face neglect by families and partners. Environmental factors such as having a high proportion of peers or friends who are teen parents, and home delivery of the first baby [25, 27] influence recurrent teenage pregnancies and teenagers with recurrent pregnancies may face challenges such as stigmatization, accused of infidelity, and hostile attitude by some health care providers [21, 26].

## Methodology

### Study design and setting

The study is a phenomenological qualitative study supported by Edmund Husserl’s philosophy [28].Informed by Husserl’s philosophy, we uncover the factors influencing recurrent teenage pregnancies and the challenges faced by the teenage mothers while they described their experiences in their lived world. During the phenomenological conversations, we ensured bracketing (a process of setting aside personal beliefs, refraining from personal judgements, and remaining open- minded while the findings unfold). The study was conducted in Domeabra, a suburb of Winneba, the capital town of Effutu Municipality in the Central Region of Ghana. The community was purposefully selected because it is a densely populated community whose members are predominantly low-income earners. The people are mainly fisher folks and these are factors that put them at a higher risk for recurrent teenage pregnancy [29].

### Study population

The population for this study was adolescents between the ages of 15 to 19 years old who either had a child or were expecting a second and or who already had more than a single child. This age range was selected because literature revealed that adolescent birth rate among persons aged 10–14 years, a very small proportion of births occur below the age of 12, and there is scarcity of data on the occurrence of childbirths amongst girls between the ages of 10 to 14 years [9, 30]. Even for females aged 15–19 years, it is less likely for first births to occur before the age of 15 years [30] and teenage pregnancy is commonly reported in females between the ages of 15–19 years who have given birth or pregnant with their first child [30]. Hence, the study purposively selected the age range of 15–19 years to assist gain adequate responses for teenagers with recurrent pregnancies.

### Sample size and sampling techniques

Mason [31] indicated that the sample size for a qualitative study ought to be a minimum of 5–25 cases as it would be enough to acquire ample data. Hagaman and Wutich [32] posit that larger sample sizes ranging from 20 to 40 participants may help reach data saturation. As larger sample is needed if saturation is needed to understand or explain complex phenomena [33]. However, Ritchie et al. [34] suggested that researchers conducting individual interviews should not conduct more than 50 interviews so that researchers can manage the complexity of the analytic task. Hence, to ensure that the researchers effectively manage the complexity of analytic take, achieve data saturation, gain more understanding and collect further examples, forty (40) participants were recruited for the study. The purpose of the study was clearly explained to them and those willing to participate were included in the study. Using convenient and snowball sampling, participants were selected for data collection.

### Data collection

Face-to-face in-depth interview with the help of an interview guide was used to collect data for the study. The study instrument consisted of three sections, A,B and C. Section A assessed the socio-demographic characteristics of participants, sections B and C assessed factors influencing recurrent teenage pregnancy and problems faced by teenagers with recurrent pregnancy respectively. Without listing of houses, the first house in the selected community was entered, the purpose of the study was explained to members of the household, all qualified participants in the household willing to participate in the study were interviewed, and members of the household were asked to refer researchers to teenagers in the community who qualified to participate in the study. After the initial house was visited, the subsequent house was visited and this was followed until the desired sample size was achieved. Participants were interviewed in their homes but some were interviewed at the nearby beach in the event that there was no privacy in the home. The selection of interview venue was dependent on the participant’s preference.

The interview was conducted in “Fante”, “Twi” dialects, and the English Language depending on the participants’ preferences. During the interview, field notes were taken, and interviews were tape-recorded with the consent of participants. Data was collected between 1st May, 2021 and 1st July, 2021 in the Domeabra Community of the Winneba Municipality, and each interview lasted for about 40 to 45 min. Data saturation was achieved after interviewing 30 participants and analysing 30 transcripts. However, to obtain more examples, and better understanding of the experiences of the teenage mothers with recurrent pregnancies, the researchers further interviewed 10 more participants. Overall, 46 participants were approached but 6 refused participation and they were not coerced to participant in the study.

### Data analysis and presentation

The study adopted Braun and Clark [35] approach to thematic analysis. Data collection and analysis were done coherently. Field notes and audio recordings from interviews were transcribed verbatim in the language of recording (Twi or Fante) and translated to English if the interview was not conducted in English. Two (2) of the researchers read through the transcripts several times to familiarise themselves and gain a general understanding of the data set. This was followed with a line-by-line coding of all transcripts, which involved a thorough reading of transcripts, and highlighting all text that on first impression appears to depict factors influencing recurrent teenage pregnancy, and problems confronted by teenagers with recurrent pregnancies. This was then used to identify sub-themes and themes for the study. The two researchers compared notes, discussed any discrepancies in their findings, and reached a consensus. A third researcher reviewed all coded data extracts for each theme, whether themes and sub-themes accurately reflected the meaning depicted by the entire data set, selected illustrative quotations, and identified any aspects of the data set that were unclear or missing. Finally, all researchers discussed and agreed on themes, sub-themes, and relevant quotes. Quotations were used in the data presentation. Table 2 presents the themes and sub-themes that emerged from the data. To ensure trustworthiness, participation in the study was voluntary [36], and there was frequent peer debriefing. The study also deployed member checking [37], thus, findings of the study were also shared with participants to validate its accuracy and whether it resonates with their experiences, and no significant revisions were implemented. The recommendations of the consolidated criteria for reporting qualitative research (COREQ) checklist were followed [38].

## Findings

### Socio-demographic characteristics

The result of the study revealed that the age of participants spanned from 16 to 19 years and the frequent age of first pregnancy was 16 years (32.5%). It might be noted that none of the study participant was 15 years. The number of children pertaining to a mother ranged from 2 to 3. The majority (47.5%) of the teenage mothers had attained Senior High School Education and they were single (80%). 72.5% of the participants were unemployed and of those who were employed, most of them were traders (64%). Table 1 depicts these results.

**Table 1:** Socio-demographic characteristics of participants

Characteristic	N	%	Characteristics	N	%
***Age***			***Living with parents***		
16	1	2.5	Yes	28	70
17	3	7.5	No	12	30
			Total	40	100
18	9	22.5	***Number of children***		
19	27	67.5	2	38	95
Total	40	100	3	2	5
***Ethnicity***			Total	40	100
Akan	22	55	***Age at first pregnancy***		
Ewe	11	27.5	13	1	2.5
Ga–Adangbe	6	15	14	7	17.5
Hausa	1	2.5	15	8	20
Total	40	100	16	13	32.5
***Religious affiliation***			17	11	27.5
Christianity	36	90	Total	40	100
Islam	4	10	***In school before pregnancy***		
Total	40	100	Yes	31	77.5
***Level of education***			No	9	22.5
Junior High	10	25	Total	40	100
Senior High	19	47.5	*Dropped out after the pregnancy*		
Vocational	1	2.5	Yes	18	58
Tertiary	10	25	No	13	42
Total	40	100	Total	31	100
***Marital status***			*Dropped out of school at;*		
Single	32	80	Junior High School	8	44
Married	2	5	Senior High School	10	56
			Total	18	100
Cohabiting	6	15			
Total	40	100			
***Married after pregnancy***					
Yes	2	5			
No	38	95			
Total	40	100			
***Employed***					
Yes	11	27.5			
No	29	72.5			
Total	40	100			
Type of employment*					
Trading	7	17.5			
Hairdresser/Beautician	2	5			
Seamstress	1	2.5			
Storekeeping	1	2.5			
Total	11	27.5			

### Themes and sub-themes of the study

The findings comprise the inferred views of the teenage mothers recruited for the study. The views of the teenage mothers on factors influencing recurrent teenage pregnancies and the problems confronting these teenage mothers were the constructed categories in the data gathered. The constructs established and explained in the interviews were organised into themes and sub-themes as presented in the Table 2. The study identified five sub-themes for factors influencing recurrent teenage pregnancy, and two sub-themes for problems confronting teenagers with recurrent pregnancies.

**Table 2:** Themes and sub-themes on factors influencing recurrent teenage pregnancy and problems of recurrent teenage pregnancy

Themes	Sub-themes
Factors influencing teenage pregnancy	Poverty
	Peer pressure
	Parental neglect
	Living with a partner
	Inadequate knowledge of contraceptives
Problems confronted by teenagers with recurrent teenage pregnancy	Financial problems
	Stigmatization

### Factors influencing recurrent teenage pregnancy

**Fig. 1:**
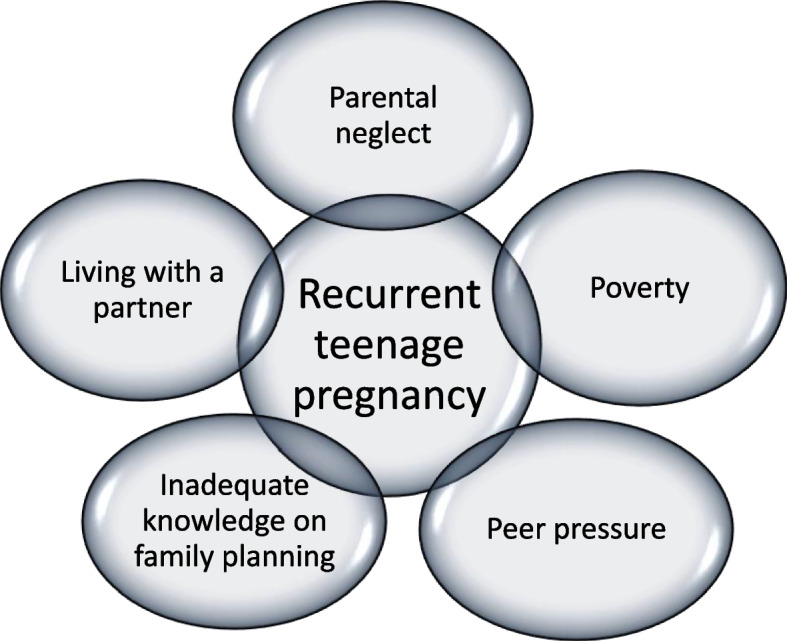
Factors influencing recurrent teenage pregnancy.Source: Authors’ contruct (July 2021)

The study discovered the following factors influencing the occurrence of recurrent teenage pregnancy. Figure 1 presents this finding.

#### Peer pressure

The study discovered that peer pressure was one of the factors that gave rise to recurrent pregnancies amongst teenagers. Participants expressed that childbirth mirrored a female’s fertility, and the humiliation of not having children led to their pregnancies. Participants were of the view that their peers mocked them for not having children, and their peers disapproved of using modern contraceptives as it is perceived as a cause of barreness which influenced them to discontinue the use of family planning methods resulting in pregnancies.

A participant said:

“There was a time when my friends and I gathered around to just have a usual talk and they started teasing me, saying that I could never have children because I always took the emergency contraceptive pill after sex so I wanted to prove them wrong that I was fertile and that was how I ended up being pregnant.” (participant 2).

#### Parental neglect

The results of the study identified that parental neglect also contributed to recurrent teenage pregnancies. Participants discussed how they involved themselves in sexual activities due to lack of parental support after their first birth. Participants expressed that their parents do not provide adequate monitoring of their day-to-day activities, thus, they do as they praised and after their first children, they were treated as outcast by their parents.

A participant revealed:

“My parents neglected me after I gave birth to my first child. They behaved as if they didn’t know me. In the first place, it was because of them that was why I was engaging in sexual activities because there was improper parental monitoring.” (participant 31).

#### Poverty

Poverty also contributed to recurrent teenage pregnancies. A number of adolescent mothers expressed how poverty contributed to their involvement in sexual activity. The need for money was what influenced teenagers to get involved in sexual activities. Participants with financial difficulties engaged in sexual activities exposing them to recurrent pregnancies. They expressed the need to exchange sex for money to feed themselves and their children, and to be able to afford their basic needs.

A participant said:

“I needed money to buy certain petty things needed by a normal girl and my parents could not provide for me so at that point I was really in need of money so I met this guy who said he would give me money if I had sexual intercourse with him” (participant 11).

#### Living with a partner

The findings of the study revealed that participants who cohabited with their male partners and those who got married and lived with their partners were susceptible to recurrent pregnancies. When teenagers live with their male partners, either customarily married or not, they engage in sexual intercourse. Some teenage mothers expressed how their first birth led to early marriage, and moving to live with their partners contributed to recurrent pregnancies.

A participant narrated:

“I was living with my parents after I gave birth to my firstborn, but my parents were of the view that I should get married to my boyfriend and that was how I got married. After the marriage, I engaged in sexual activity with my husband and that was how I got pregnant again” (participant 6).

#### Inadequate knowledge of family planning

It was revealed by the findings of the study that teenage girls had inadequate knowledge on family planning. Their inadequate knowledge and understanding of the use and beneficial effect of the different family planning types/methods lead to erroneous beliefs about family. Most of the participants held misconceptions about the scientific methods of family planning thus, they preferred not to use contraceptives. They narrated how they perceived contraceptives had side effects,

A participant said:

“I didn’t use contraceptives because I thought it would have an impact on my body if not immediately then somewhere in the future. I am saying this because I have a friend who complains of gaining weight anytime she takes in postinor-2.” (participant 26).

### Problems encountered by teenagers with recurrent pregnancies

The findings of the study indicated that financial problems and stigmatisation were challenges faced by the teenage mothers.

#### Financial Problems

Participants expressed that they encountered financial difficulties because their either parents or partners abandoned them. They said that they were unemployed and sometimes their partners too were unemployed. Even if their partners were employed, their salaries were nothing good. In some cases, partners denied the paternity of infants due to anger.

A participant expressed:

“The father of my first child is different from that of the second and I even find it difficult to cater for the children since their fathers are not working and they are irresponsible. When I was even pregnant with the second child, his father tried denying the baby was his and even insulted me of being a prostitute.” (participant 4).

Another participant narrated:

“Sometimes, it’s very hard for me to feed my children since I’m not working and no one in my family cares.” (Participant 37).

Another teenage mother expressed:

“Getting money to cater for ourselves is not an easy task since my parents drove me out of the house to go and live with my partner. My partner also doesn’t have enough money to care for us because he works as a carpenter and the earning is not all that good.” (participant 27).

#### Stigmatisation

Stigma is another challenge confronted by teenage mothers. Participants were mostly stigmatised because their partners abandoned them. Participants expressed that they were gossips about them in their communities, and name calling such as ‘bad girls’.

A teenage mother expressed:

“They were insulting me, especially the old women. They were saying instead of me going for a well-established man, I rather went in for small–small boys who would later abandon me. I was really ashamed after hearing this” (participant 36).

Another participant added:

“I was stigmatised and others kept asking if the second child was for the same man I had the first with to the extent that some even called me a prostitute.” (participant 3).

## Discussion of Findings

The results of the study revealed that the participants were between the ages of 16 and 19 years with the frequent age of first pregnancy being 16 years. This is because at this age, the young adult will like to experiment sexual intercourse due to psychological and social factors coupled with inadequate knowledge on sexual and reproductive health [39]. However, none of the participants was 15 years. This was in line with the finding of [31]. The authors posit that for females aged 15–19 years, there is less likelihood for the first birth to occur before the age of 15 years. Thus, even if a female is 15 years at the delivery of her first baby, before her second baby, she would be 16 years. The results of the study further revealed that majority of the participants had a low level of education, specifically, secondary level of education. The teenage mother has to cater for herself and her young ones, hampering her ability to attain a high level of education. This low level of educational attainment does not assist the individual acquire a qualification or skill to be gainfully employed and obtain an adequate salary, thus, impeding the mother’s ability to provide for herself, and her children. The results of this current study also revealed that majority of the participants were Christians and a few were Muslims. This is in line with the finding of the [40] in which the majority of the residents in Effutu Municipality were Christian. It was also observed that the majority were single but reproducing. These can be related to the fact that in the current Ghanaian communities, foreign cultures such as Christianity and Islam have been borrowed and have abandoned their traditional means such as puberty rites that were used to curb premarital sex and unwanted teenage pregnancies [41, 42].

The findings of the study further revealed that peer pressure was a factor influencing recurrent pregnancies amongst teenagers. Quist-Adade [43] acknowledged that peer influence from friends and classmates is a factor influencing recurrent pregnancies amongst teenagers. Occasionally, teenagers’ voluntarily get pregnant to gain respect from their peers [20] and prove their fertility, as motherhood is an ultimate goal of the Ghanaian woman [44]. However, if a teenager gets pregnant for the first time, it predisposes her to recurrent teenage pregnancies [39].

Poverty also contributed to recurrent pregnancies amongst teenagers. Teenagers engage in transactional sex as a means of getting money to fend for themselves and their children. Aslam et al. [10] and Krugu et al. [19] acknowledged that the majority of girls indulge in sexual relationship primarily for economic gains. Hence, when teenage mothers have financial difficulties in feeding themselves and their children, they are likely to get involved in sexual activities that could lead to recurrent pregnancies.

The findings of the study further indicated that parental neglect is a factor influencing recurrent teenage pregnancy. This finding is consistent with that of Govender, Naidoo, and Taylor [45]. Support from family members play a pivotal role in curbing recurrent teenage pregnancy. This can be attributed to the fact that family members can support the teenager to meet her needs, deterring her from further engaging in sexual activities.

Living with a partner was revealed in our study to contribute to recurrent teenage pregnancies. Ngoda et al. [27] indicated that marriage is a factor that influences recurrent teenage pregnancy because married couples live together, encouraging unprotected sexual intercourse and consequent, recurring pregnancies for the teenager.

Inadequate knowledge on family planning was also identified as a contributor to recurrent teenage pregnancies. Albuquerque et al. [46] and Ngoda et al. [27] acknowledged that there is a low prevalence of recurrent pregnancies amongst teenage mothers who use contraceptives, as family planning protects mothers from unplanned pregnancies.

Financial difficulties and stigmatisation were the challenges faced by teenagers with recurrent pregnancies. Talungchit et al. [47] indicated that teenage pregnancy mitigates the chance to acquire higher education which is a paramount predictor to financial difficulties and poverty. As the teenage mother is not gainfully employed, it is obvious she will have no stable source of income, therefore, the high tendency of facing financial difficulties. Mogan et al. [39] indicated that poverty is both a predictor and a consequence of teenage pregnancy. Additionally, in the Ghanaian society, marriage confers on a woman a high degree of respectability [48]. Marriage is seen as a permit for reproducing and coupled with the teenagers’ inability to prove paternity of her children in a cultural milieu where parenting is defined by emphasizing the role of a father [49], it is certain she would face stigmatisation in society.

### Strengths and limitations of the study

This study is the first phenomenological qualitative study investigating the factors influencing the recurrent teenage pregnancy and challenges confronted by teenagers with recurrent pregnancies in the central region and Ghana as a whole. Furthermore, the study has provided in-depth insight into the factors influencing recurrent teenage pregnancy to guide the implementation of community-based interventions and future studies. The study however had limitations. The study was conducted among ages 15- 19 in the Effutu municipality of the central region. Hence, the findings described here may not reflect the perspectives of teenagers with recurrent teenage pregnancies in other regions of Ghana with different socioeconomic circumstances. The findings of the study should be generalized with care. Future studies should focus on the possible occurrence of recurrent teenage pregnancies among ages 10–14 and in other regions to help provide a national data for a nationwide intervention to curb recurrent teenage pregnancies.

## Conclusion

The results of the study revealed that recurrent teenage pregnancy occurs in teenagers within the age ranges of 16 to 19 years in the Effutu Municipality. Majority of these teenagers had attained only a secondary level of education and they were unemployed. Recurrent teenage pregnancy is multifaceted. Factors such as peer pressure, poverty, living with a partner, parental neglect and inadequate knowledge on family planning promote the occurrence of recurrent teenage pregnancies.

Teenage mothers are confronted with financial difficulties and stigmatisation in society. Hence, there is the need to intensify family planning education amongst teenagers, especially, among teenage mothers, educate the general public on good parental practices, the harmful effects of stigmatisation on the teenage mother and her off-springs, and create a social support network for teenagers with recurrent pregnancies to help curtail this public health menace.

## Data Availability

The data that support the findings of this study are available from corresponding author [Agartha Afful Boateng] with participants’ written consent but restrictions apply to the availability of these data to the public. Due to ethical guidelines followed by the study and it could compromise research participant privacy and or consent.
